# Supplementary material for: Predictors of Adverse Cardiovascular Events After CABG in Patients with Previous Heart Failure
Source: Life (Basel). 2025 Feb 28;15(3):387. doi: 10.3390/life15030387 (PMC11944089; doi:10.3390/life15030387)
Supplement: Supplementary file 1 [file life-15-00387-s001.zip › life-3466289-supplementary.pdf]

Table S1. Echocardiographic parameters of patients before CABG

**Parametrs**	**Group 1 (*n*=45)**	**Group 2 (*n*=37)**	**p value**
LA, mm	42 (40; 44)	47 (41; 52)	0.002
EDD, mm	51 (49; 56)	60 (51.5; 66.5)	0.001
ESD, mm	34.5 (32; 43)	49 (35.5; 55.5)	<0.001
EDV, mL	118 (108.5; 155)	188 (122; 238.5)	<0.001
ESV, mL	47 (39.5; 86.5)	117 (49.5; 160)	<0.001
	n=39	n=30	
E/A, units	1 (0.82; 1.12)	1.5 (0.74; 2.44)	0.101
E/e', units			
EDI, mL/m2	62.6 (57.25; 85.55)	94.5 (64.75; 116.2)	0.001
ESI, mL/m2	24.3(20.95; 43.8)	63.1 (25.65; 78.85)	0.001
SV, mL	68 (63; 72.75)	67 (56; 77.5)	0.849
MMLV, g			
IMMLV, g/m2	96 (90; 104)	106 (94.2; 120)	0.022
MVreg, grade	1 (0.5; 1)	1 (1; 2)	0.003
AVreg, grade	0 (0; 1)	0 (0; 0.75)	0.215
TVreg, grade	0 (0; 0.875)	0 (0; 1)	0.023
	n=18	n=23	
PAPs, mmHg	30 (27.75; 38.5)	45 (30; 53)	0.021
Hypokinesis or akinesis zone, n (%)	21 (46.7)	9 (24.3)	0.008
Cardiac aneurysm, n (%)	6 (13.3)	10 (27)	0.119
LVEF %	60 (44; 64)	37 (28.5; 60)	0.001
LVEF: less than 50%	16 (35.6)	25 (67.6)	0.004

LA – Left atrium, EDD - End diastolic diameter, ESD - End systolic diameter, EDV - End diastolic volume, ESV - End systolic wolume, EDI - End diastolic index, ESI - End systolic index, SV - Stroke volume, MMLV - left ventricular myocardial mass, IMMLV - left ventricular myocardial mass index, MVreg – degree of mitral valve regurgitation, AVreg - degree of aortic valve regurgitation, TVreg - degree of tricuspid valve regurgitation, PAPs - pulmonary artery systolic pressure, LVEF - left ventricular ejection fraction. Continuous variables were presented as median and interquartile range (Me (Q25; Q75). Categorical data were presented in absolute and relative values: n (%).
